# Best practices for avoiding dominant experimental bias in analysis of multielectrode array signals

**DOI:** 10.1186/1471-2202-15-S1-P208

**Published:** 2014-07-21

**Authors:** Tyler Stone, Uzair Khawaja, Nardeen Perko, Thomas R Kiehl, Charles Bergeron

**Affiliations:** 1 Analytics Lab, Albany College of Pharmacy and Health Sciences, Albany, NY, 12208, USA; 2 Neural Stem Cell Institute, Rensselaer, NY, 12144, USA

## 

Multielectrode arrays (MEA) are broadly used for in vitro cell culture observation. Complete realization of this platform’s potential to the study of disease and development depends on the capability to make 1) reliable and repeatable observations, 2) neuronal network behavior characterizations, and 3) meaningful comparisons between cultures. Using an extensive, previously published, set of recordings [3] we demonstrate that naïve processing of this data leaves us vulnerable to bias from measurement error.

**Figure 1:**
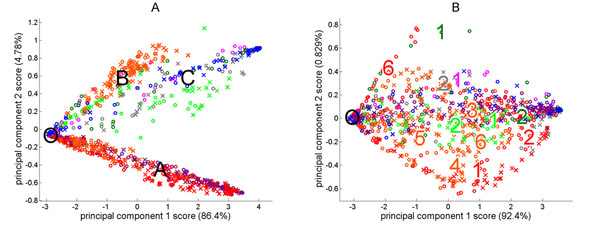
**(A)** Principal components analysis biplots using all 60 electrodes. Each marker is a recording. Colors indicate batches: **Batch 1 (purple)**, **Batch 2 (red)**, **Batch 3(orange)**, **Batch 4 (light green)**, **Batch 5 (grey)**, **Batch 6 (blue)**, **Batch 7 (pink)**, and **Batch 8 (dark green)**. The large black circle is the dense core of recordings showing weak spike activity. Classes A, B and C are also labeled. **(B)** PCA biplots using the 47 electrodes following deletion of biased ones. Some regions are associated with specific cultures identified by culture numbers and batch colors.

We utilize 878 recordings from embryonic rat cortex cell cultures collected from 60-electrode, grid-type (200 μm) MEA’s. We modeled each recording as a 60-node directed weighted graph with weights describing electrode connectivity and nodal clustering coefficients [1] as features. Principal components analysis (PCA) reduced the dimensionality of this feature space [2]. We observe a dense core of recordings showing weak spike activity, and 3 classes defined by batch (labeled in Figure 1A). Surprised at this structure, we sought a non-biological explanation. We identified 13 defective or biased electrodes as sources of systematic measurement error. Removing the affected electrodes produced a more complex interplay of inter- and intra-batch variability (Figure 1B).

Bias from defective electrodes can be avoided wholly by proper documentation of experimental conditions. This highlights the need for best practices when recording MEA signals. Our analysis highlights possibilities for post-experimental review to identify unknown issues and retroactively handle them. Thereupon, we make a case for transparency in data reporting and propose best practices for experimental and analysis phases.
